# A novel synthetic oleanane triterpenoid, 2‐cyano‐3,12‐dioxoolean‐1,9‐dien‐28‐oic acid, regulates mechanical allodynia by rescuing neuronal cell death and glial cell activation in the spinal cord of resiniferatoxin‐treated rats

**DOI:** 10.1002/brb3.3398

**Published:** 2024-02-04

**Authors:** Ying‐Yi Lu, Chia‐Yang Lin, Hung‐Pei Tsai, Chih‐Lung Lin, Chieh‐Hsin Wu

**Affiliations:** ^1^ Department of Dermatology Kaohsiung Veterans General Hospital Kaohsiung Taiwan; ^2^ Department of Post‐Baccalaureate Medicine, School of Medicine, College of Medicine National Sun Yat‐sen University Kaohsiung Taiwan; ^3^ Shu‐Zen Junior College of Medicine and Management Kaohsiung Taiwan; ^4^ Department of Nuclear Medicine Kaohsiung Medical University Hospital Kaohsiung Taiwan; ^5^ Division of Neurosurgery, Department of Surgery Kaohsiung Medical University Hospital Kaohsiung Taiwan; ^6^ Department of Surgery, School of Medicine, College of Medicine Kaohsiung Medical University Kaohsiung Taiwan; ^7^ Center for Big Data Research Kaohsiung Medical University Kaohsiung Taiwan; ^8^ Drug Development and Value Creation Research Center Kaohsiung Medical University Kaohsiung Taiwan

**Keywords:** 2‐cyano‐3,12‐dioxoolean‐1,9‐dien‐28‐oic acid, mechanical allodynia, resiniferatoxin, triterpenoid

## Abstract

**Background:**

Treating postherpetic neuralgia (PHN), which is characterized with a long‐lasting lancinating mechanical allodynia or hyperalgesia, is a big challenge as it is hard to achieve complete resolution. A synthetic triterpenoid, CDDO (2‐cyano‐3,12‐dioxoolean‐1,9‐dien‐28‐oic acid) can exert pleiotropic effects including anti‐inflammation and neuroprotective activities. Nevertheless, the antinociceptive effect of CDDO and its derivatives remains unknown. Resiniferatoxin (RTX) is easily feasible, and an RTX‐treated rodent model can mimic the PHN‐like symptoms. Therefore, RTX‐treated rats were used to serve as a PHN rats’ model in the study to elucidate whether a synthetic triterpenoid, CDDO, can improve mechanical allodynia in RTX‐treated rats.

**Methods:**

The antinociceptive effects of CDDO were assessed by behavioral tests, western blotting, and immunohistochemistry. Paw withdrawal mechanical threshold was determined using calibrated forceps.

**Results:**

Administration of RTX led to mechanical allodynia, neuronal cell death, and glial cell activation in the spinal cord of RTX‐treated rats. A synthetic triterpenoid, CDDO, blocked RTX‐induced mechanical allodynia, rescued neuronal cell death, and inhibited glial cell activation in the spinal cord of RTX‐treated rats.

**Conclusions:**

Our study provides a novel result that a synthetic triterpenoid, CDDO, can interfere neuronal cell death and glial cell activation in the spinal cord of RTX‐treated rats. Hence, CDDO is an alternative therapeutic choice for PHN.

## Graphical abstract

Resiniferatoxin‐treated rats elicit mechanical allodynia. A novel synthetic oleanane triterpenoid, 2‐cyano‐3,12‐dioxoolean‐1,9‐dien‐28‐oic acid, can improve mechanical allodynia by interfering neuronal cell death and glial cell activation in the spinal cord of resiniferatoxin‐treated rats.

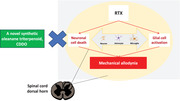

## 1 INTRODUCTION

Reactivation of latent varicella zoster virus in sensory ganglia leads to grouped cutaneous blisters and neuralgia along the corresponding sensory nerves (Schmader, 2018). After the skin eruption resolved, postherpetic neuralgia (PHN) characterized with a lancinating mechanical allodynia or hyperalgesia could last beyond 3 or more months (Zorzoli et al., 2018). The disproportionate painful sensation without corresponding tissue damage is its characteristic (Oaklander, 2008). Increasing with growing age, the incidence of PHN even rises to 75% in people aged 70 (Delaney et al., 2009). The deficiency of myelin and axon, dorsal horn atrophy, and change of epidermal axon density contribute to the peripheral and central sensitization after receiving nociceptive stimulus (Chen et al., 2017; Watson et al., 1991; Werner et al., 2017). Treating PHN is a big challenge as it is hard to achieve complete resolution even after multimodal therapy and it often impairs life quality due to several complications (Gruver & Guthmiller, 2023; Schutzer‐Weissmann & Farquhar‐Smith, 2017).

As the chemical structure of triterpenoids resembles those of steroids, triterpenoids’ extracts exhibit anti‐inflammatory, antioxidant, antimicrobial, antidiabetic, as well as cardio‐ and hepatoprotective activity (Phillips et al., 2006). To augment the natural triterpenoids’ biological functions, 2‐cyano‐3,12‐dioxoolean‐1,9‐dien‐28‐oic acid (CDDO) and its derivatives were synthesized to amply their pleiotropic capability depending on different doses (Borella et al., 2019; Robles et al., 2016). They exhibit anti‐inflammatory and antioxidative effects at low doses, induce cell differentiation at intermediate doses, lead to anti‐proliferative, cytotoxic, and proapoptotic activities at high doses (Borella et al., 2019). Several studies have documented that CDDO and its derivatives have neuroprotective effects. They can exert cytoprotective properties in neurons, astrocytes, and microglia by activating NADPH quinone oxidoreductase 1 and reducing nitric oxide synthesis (Graber et al., 2011). Activation of glial cells and neuronal cell death contribute to neuropathic pain (Hains & Waxman, 2006; Ohtori et al., 2004). CDDO and a derivative with a methyl ester at C‐28 can reduce microglial activities and preserve neuronal cells survival (Suh et al., 1999; Tran et al., 2008). Nevertheless, the antinociceptive effect of CDDO and its derivatives remains unknown.

Resiniferatoxin (RTX) is a capsaicin ultrapotent analog, which can bind to transient receptor potential vanilloid 1 (TRPV1) irreversibly. It can lead to unmyelinated afferent neurons degeneration, myelinated afferent fibers damage, abnormal nerve sprouting into the lamina II of the spinal dorsal horn, and denervation of skin in the same affected dermatome (Hsieh et al., 2008). It generates paradoxical changes in mechanical and thermal sensitivities, mimicking the distinctive PHN symptoms (Pan et al., 2003; Yuan et al., 2017). Therefore, series studies have showed that systemic RTX acts as a nonviral PHN model to investigate the action of nociceptive afferents (Hong et al., 2018; Lei et al., 2016; Wu et al., 2021). As RTX is easily feasible and an RTX‐treated rodent model can mimic the PHN‐like symptoms, RTX‐treated rats were utilized to serve as a PHN rats’ model in the study. We hypothesized that a synthetic triterpenoid, CDDO, can improve mechanical allodynia in RTX‐treated rats. Later, the life of neurons and activity of glial cells were altered in the spinal cord. This study indicates that a synthetic triterpenoid, CDDO, can interfere neuronal cell death and glial cell activation in the spinal cord of RTX‐treated rats, which offers a potential choice to treat the debilitating PHN.

## 2 MATERIALS AND METHODS

### 2.1 Animals

In the experiment, adult, male, Sprague‐Dawley rats (weight of 300–350 g) were used. These rats were purchased from National Animal Center of Taiwan and kept in different cages under a controlled environment (room temperature maintained at 20–24°C) with easily accessible food and water in a 12:12‐h light‐dark cycle. All procedures in the experiment were confirmed and approved by the Animal Care and Use Committee of Kaohsiung Medical University.

### 2.2 Establishment of PHN rat model

The PHN rats’ model was established following the procedures described by Hsieh et al., and our previous experiment, which a single dose of RTX (50 ug/kg, Sigma), was administrated intraperitoneally (i.p.) (Hsieh et al., 2012, 2008; Lin et al., 2013; Wu et al., 2021). CDDO was dissolved in the DMSO to reach 50 mg/mL at concentration before use. After RTX used, CDDO‐RTX‐rats received CDDO (10 mg/kg, i.p.) every day. Control rats did not receive both RTX and CDDO. The investigators were unaware of the treatment conditions.

### 2.3 Behavioral tests

The withdrawal thresholds of hind‐paw in response to the force applied by calibrated forceps (Biosebs) were used to estimate mechanical allodynia. An abrupt withdrawal of hind paw was regarded to be a positive response. The measurements were performed before, at the third day and seventh days after treatment with CDDO. The investigators were unaware of the treatment conditions.

### 2.4 Western blotting

After rats were terminally anesthetized using Zoletil (50 mg/kg), the fourth to fifth lumbar segments of spinal cord were quickly removed and homogenized in lysis buffer containing proteinase inhibitor. To collect the supernatant, the homogenates were centrifuged at 4°C for 30 min at 13,000 rpm. After the protein concentration was estimated by a BCA Protein Assay Kit, proteins were loaded onto 8%–12% SDS‐polyacrylamide gels. The proteins were transferred onto a polyvinylidene fluoride membrane. Membranes were blocked with 5% skim milk for 1 h and incubated at 4°C overnight with following primary antibody: Akt (1: 500; Cell Signaling Technology; #9272; RRID: AB_329827), PKC‐δ (1: 500; BD; 610398; RRID:AB_397781), phospho‐Akt (Ser473) (1: 500; Cell Signaling Technology; #9271; RRID:AB_329825), and β‐actin (1: 10,000; Millipore; MAB1501R; RRID:AB_2223041). After washed with Tween 20 and Tris‐buffered saline, membranes were incubated at room temperature for 1 h with horseradish peroxidase‐conjugated secondary antibody. The immunoreactive bands were visualized by an ECL reagent and measured by MiniChemi chemiluminescent imaging and analysis system (Sage Creation Science). The intensity of bands was normalized to the loading control (β‐actin).

### 2.5 Immunohistochemistry

After rats were terminally anesthetized using Zoletil (50 mg/kg), the fourth to fifth lumbar segments of spinal cord were removed, postfixed in 4% paraformaldehyde and cut into 8 μm‐thick sections in a cryostat. After rising with phosphate‐buffered saline, blocked with 1% normal goat serum in 0.1% Triton X‐100 and 0.1% phosphate‐buffered saline at room temperature for 1 h, these sections were stained with following antibodies at 4°C for 24 h: glial fibrillary acidic protein (GFAP) (1:400; Sigma; G3893; RRID: AB_477010), NeuN (1:400; Millipore; MAB377; RRID:AB_2298772), OX‐42 (1:100; BIO‐RAD; MCA275G; RRID:AB_321301), phospho‐Akt (Ser473) (p‐Akt) (1:200; Cell Signaling Technology; #9271; RRID:AB_329825), and PKC‐δ (1:200; BD; 610398; RRID:AB_397781) for immunofluorescence. These stained sections were labeled with a mixture of Alexa Fluor 594‐conjugated anti‐mouse secondary antibody (1:500; Jackson ImmunoResearch; 115‐585‐146; RRID: AB_2338881) or Alexa Fluor 488‐conjugated goat anti‐rabbit secondary antibody (1:500; Jackson ImmunoResearch; 111‐545‐144; RRID: AB_2338052) for 1 h. TUNEL kit (Roche; 11684795910) was used to stain apoptotic cells. After washed with phosphate‐buffered saline, these stained sections were covered onto slides and examined using an Olympus fluorescence microscope. ImageJ software was used to measure the amounts of apoptotic neurons, the GFAP intensity, and the OX‐42 intensity.

### 2.6 Statistically analysis

All data in the experiment was recorded as the mean ± SEM. The differences were analyzed using Student *t* test if only two datasets or using ANOVA test followed by a Bonferroni test if multiple datasets. Besides, a *p* value less than .05 was regarded as statistically significant.

## 3 RESULTS

### 3.1 Effect of a synthetic triterpenoid, CDDO, on mechanical allodynia in RTX‐treated rats

**FIGURE 1:**
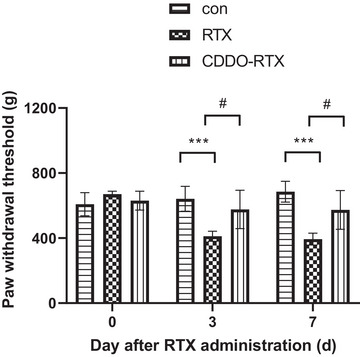
Effect of a synthetic triterpenoid, 2‐cyano‐3,12‐dioxoolean‐1,9‐dien‐28‐oic acid (CDDO), on mechanical allodynia in resiniferatoxin (RTX)‐treated rats. Mechanical threshold was evaluated through paw withdrawal threshold before, at the third day and seventh days after treatment with CDDO in RTX‐treated rats. CDDO significantly increased the mechanical threshold until 7 days later. (*n* = 6) Mean ± SEM is shown for data (***p < .001 vs. control (con) group, #*p* < .05 vs. CDDO‐RTX group).

To assess the effect of CDDO on mechanical allodynia in RTX‐treated rats, we evaluated the paw withdrawal threshold by using calibrated forceps. The baseline mechanical threshold of every group was similar. RTX significantly decreased the mechanical threshold (*p* < .001), whereas treatment with CDDO significantly increased the mechanical threshold until 7 days later (*p* < .05) (Figure 1).

### 3.2 Effect of a synthetic triterpenoid, CDDO, on glial cell activation in RTX‐treated rats

**FIGURE 2:**
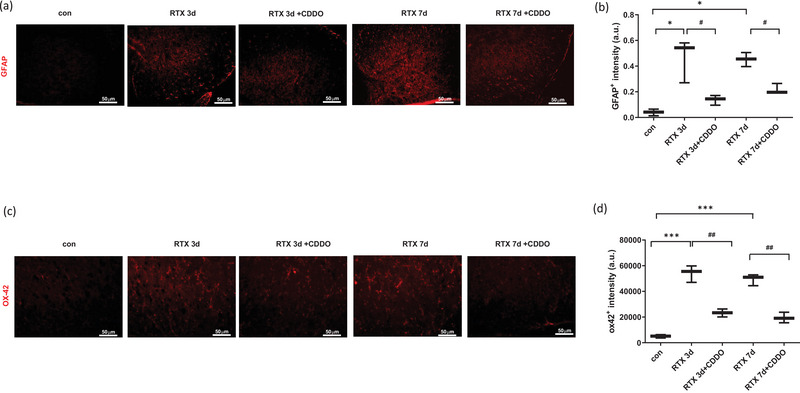
Effect of a synthetic triterpenoid, 2‐cyano‐3,12‐dioxoolean‐1,9‐dien‐28‐oic acid (CDDO), on glial cell activation in resiniferatoxin (RTX)‐treated rats. Immunohistochemistry study was undergone to stain the sections of RTX‐treated rats’ spinal cord. (a) Representative images for GFAP intensity. GFAP, red staining. (b) Quantification of GFAP intensity. (c) Representative images for OX‐42 intensity. OX‐42, red staining. (d) Quantification of OX‐42 intensity. Scale bar, 50 μm. Mean ± SEM is shown for data (*n* = 3) (*p < .05, **p < .01, ***p < .001 vs. control (con) group, #*p* < .05, ##*p* < .01 vs. CDDO‐RTX group).

To assess the effect of CDDO on glial cell activation in RTX‐treated rats, we evaluated the intensity of GFAP (astrocyte marker) and the intensity of OX‐42 (microglia marker) in the spinal cord dorsal horn by using immunohistochemistry. RTX significantly increased the GFAP intensity (*p* < .05), whereas treatment with CDDO significantly decreased the GFAP intensity until 7 days later (*p* < .05) (Figure 2a,b). Besides, RTX significantly increased the OX‐42 intensity (*p* < .001), whereas treatment with CDDO significantly decreased the OX‐42 intensity until 7 days later (*p* < .01) (Figure 2c,d).

### 3.3 Effect of a synthetic triterpenoid, CDDO, on neuronal cell death in RTX‐treated rats

**FIGURE 3:**
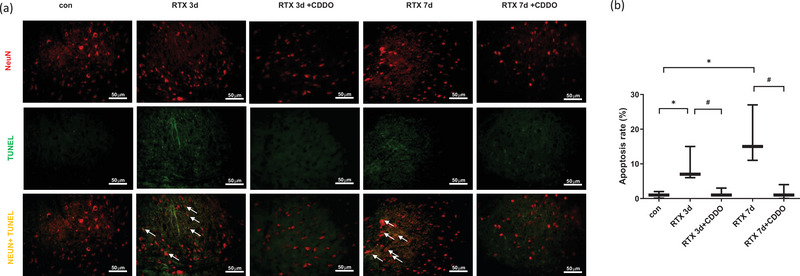
Effect of a synthetic triterpenoid, 2‐cyano‐3,12‐dioxoolean‐1,9‐dien‐28‐oic acid (CDDO), on neuronal cell death in resiniferatoxin (RTX)‐treated rats. Immunohistochemistry and TUNEL studies were undergone to stain the sections of RTX‐treated rats’ spinal cord. (a) Representative images for death of neurons. NeuN, red staining; TUNEL, green staining; merged, yellow staining. (b) Quantification of apoptotic rates. Scale bar, 50 μm. Mean ± SEM is shown for data (*n* = 3) (*p < .05 vs. control (con) group, #*p* < .05 vs. CDDO‐RTX group).

To assess the effect of CDDO on neuronal cell death in RTX‐treated rats, we evaluated the merged intensity of NeuN (neuron marker) and TUNEL in the spinal cord dorsal horn by using immunohistochemistry. RTX significantly increased the apoptotic rate of neurons (*p* < .05), whereas treatment with CDDO significantly decreased the apoptotic rate of neurons until 7 days later (*p* < .05) (Figure 3a,b). In summary, CDDO attenuated mechanical allodynia, reduced glial cell activation and neuronal cell death in the spinal cord of RTX‐treated rats.

### 3.4 A synthetic triterpenoid, CDDO, regulated PKC‐δ/p‐Akt level in the spinal cord of RTX‐treated rats

**FIGURE 4:**
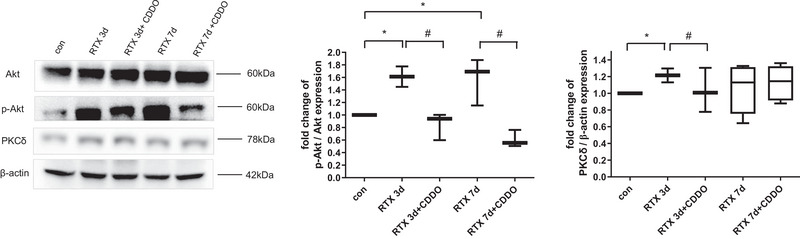
A synthetic triterpenoid, 2‐cyano‐3,12‐dioxoolean‐1,9‐dien‐28‐oic acid (CDDO), regulated PKC‐δ/phosphorylated Akt (p‐Akt) level in the spinal cord of resiniferatoxin (RTX)‐treated rats. The PKC‐δ, Akt, and p‐Akt protein levels in the spinal cord of RTX‐treated rats were measured by western blotting. Mean ± SEM is shown for data (*n* = 3) (*p < .05 vs. control (con) group, #*p* < .05 vs. CDDO‐RTX group).

**FIGURE 5:**
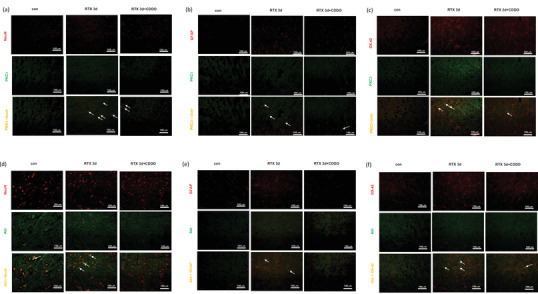
PKC‐δ and p‐AKT were expressed on neurons, astrocytes, and microglia in the spinal cord of resiniferatoxin (RTX)‐treated rats. Immunohistochemistry study was undergone to stain the sections of RTX‐treated rats’ spinal cord before, at the third day after treatment with 2‐cyano‐3,12‐dioxoolean‐1,9‐dien‐28‐oic acid (CDDO). PKC‐δ was expressed on (a) neurons, (b) astrocytes, and (c) microglia. PKC‐δ, green staining; NeuN, red staining; GFAP, red staining; OX‐42, red staining; merged, yellow staining. Scale bar, 50 μm. p‐AKT were expressed on (d) neurons, (e) astrocytes, and (f) microglia. p‐AKT, green staining; NeuN, red staining; GFAP, red staining; OX‐42, red staining; merged, yellow staining. Scale bar, 50 μm.

Next, western blotting was performed to estimate whether PKC‐δ/Akt signaling was involved in regulating mechanical allodynia. RTX significantly increased the protein level of PKC‐δ (*p* < .05), whereas treatment with CDDO significantly decreased the level until 3 days later (*p* < .05) (Figure 4). Besides, RTX significantly increased the protein level of p‐Akt (*p* < .05), whereas treatment with CDDO significantly decreased the level until 7 days later (*p* < .05) (Figure 4). Furthermore, immunohistochemistry study was utilized to clarify cellular localization regulated by PKC‐δ/Akt signaling. PKC‐δ (Figure 5a–c) and p‐Akt (Figure 5d–f) were expressed on neurons, astrocytes, and microglia of spinal cord. Through PKC‐δ/Akt signaling, CDDO can regulate neuronal cell death and glial cell activation in the spinal cord of RTX‐treated rats.

## 4 DISCUSSION

**FIGURE 6:**
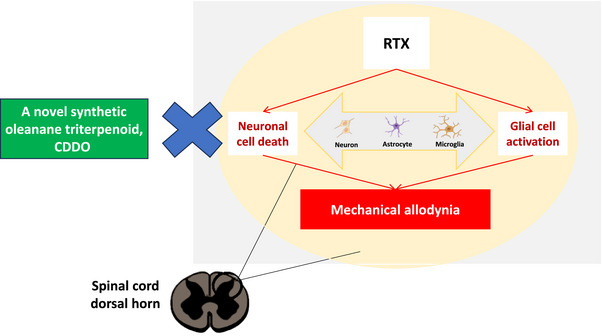
The schematic signaling pathways involved in resiniferatoxin (RTX)‐treated rats after receiving daily 2‐cyano‐3,12‐dioxoolean‐1,9‐dien‐28‐oic acid treatment (CDDO). RTX‐treated rats elicit mechanical allodynia. A novel synthetic triterpenoid, CDDO, can improve mechanical allodynia by interfering neuronal cell death and glial cell activation in the spinal cord of RTX‐treated rats.

A growing body of evidence showed that a synthetic triterpenoid, CDDO can exert pleiotropic effects including neuroprotective activities (Graber et al., 2011). However, its antinociceptive effect remains unclear. In the present study, we utilized RTX‐treated rats to serve as a PHN rats’ model. Our results demonstrated that CDDO can improve mechanical allodynia by interfering neuronal cell death and glial cell activation in the spinal cord of RTX‐treated rats (Figure 6). Therefore, our study suggested that a novel synthetic oleanane triterpenoid, CDDO, is an alternative therapeutic choice for PHN.

After nerve injury, spinal disinhibition activates central sensitization and leads to mechanical allodynia (Cao et al., 2022). The spinal cord dorsal horn is the major center to modulate pain signals, where activated glial cells release extracellular signaling molecules to trigger secondary somatosensory neurons (Chen et al., 2021). Nociception is finally transmitted to the supraspinal brain area to process the pain perception. Being the first relay center to percept noxious stimulus, spinal cord dorsal horn can exhibit excessive metabolism and activate inflammation to regulate PHN and serve as a crucial role (Zhao et al., 2019). In steady circumstance, astrocytes in central nervous system (CNS) provide nutritional and structural support for neurons, whereas microglia behaves as CNS‐resident immune cells to keep homeostasis and maintain neural function (Haydon, 2001; Kierdorf & Prinz, 2017). After nerve injury, astrocytes and microglia undergo glial cell activation with structural and functional transformation (Milligan & Watkins, 2009). Microgliosis in ipsilateral spinal dorsal horn developed rapidly followed by astrogliosis (Kohno et al., 2018). Activated central glia release inflammatory cytokines sensitize nociceptive neurons so that pathological pain persists (Ji et al., 2019). Augmented inflammatory cytokines further activate neuronal cells apoptosis to derive hyperalgesia after loss of inhibitory neurons (Hatch et al., 2018). In RTX‐treated rats, both unmyelinated and myelinated nerve fibers are damaged (Wu et al., 2013). RTX not only activates astrocytes and microglia but also increases TNFα to induce death of neurons, which leads to nociceptive hyperalgesia (Lei et al., 2016; Leo et al., 2017). In RTX‐treated rats, blockade of hepatoma‐derived growth factor (HDGF) attenuates mechanical allodynia by inhibiting spinal cord astrogliosis (Wu et al., 2021). In our study, RTX led to mechanical allodynia by activating microgliosis, astrogliosis as well as neuronal cell death, which is consistent with previous studies. CDDO attenuated mechanical allodynia by reducing glial cell activity and death of neurons in the spinal cord of RTX‐treated rats.

PKC‐δ/Akt signaling is involved in cell death (Basu & Pal, 2010; Jin et al., 2014). Regardless of PI3K, PKC‐δ regulates cancer cells survival by activating Akt (Grossoni et al., 2007) or retinal neuronal cell apoptosis of diabetic rats by activating Akt (Kim et al., 2008). Akt regulates mTOR downstream signals to induce hippocampal neuronal cell death (Liu et al., 2014). In response to noxious stimulus, PKC‐δ/Akt signaling can regulate pain processing in neuropathic pain such as paclitaxel neuropathy and diabetic neuropathy (Chen et al., 2017; He & Wang, 2015; Velazquez et al., 2007). Spared nerve injury activates PKC‐δ/Akt to aggravate neuropahic pain mediating by membrane estrogen receptors (Wright et al., 2019). Through PKC‐δ activation, capsaicin enhances TRPV1 expression to cause thermal hyperalgesia (Cesare et al., 1999). Capsaicin also activates Akt in neuronal cells of spinal cord dorsal horn to induce mechanical allodynia (Sun et al., 2006). After surgery, Akt is upregulated in microglia and neuronal cells of spinal cord in mice to elicit postsurgical pain (Xu et al., 2019). Through decreasing Akt level of spinal cord, blockade of HDGF attenuates mechanical allodynia (Wu et al., 2021). Li et al. (2021) had revealed that RTX caused hyperalgesia by activation of PKC‐δ in microglia. In our study, RTX increased the protein level of PKC‐δ/p‐Akt, whereas treatment with CDDO decreased the level (Figure 4). PKC‐δ and p‐Akt were expressed on neurons, astrocytes, and microglia of spinal cord (Figure 5). Through PKC‐δ/Akt signaling, CDDO can regulate neuronal cell death and glial cell activation in the spinal cord of RTX‐treated rats without PI3‐kinase.

## 5 CONCLUSIONS

In summary, our results show a novel finding that a synthetic triterpenoid, CDDO, is an alternative therapeutic choice for PHN. CDDO can interfere neuronal cell death and glial cell activation in the spinal cord of RTX‐treated rats.

## AUTHOR CONTRIBUTIONS

**YingYi Lu**: Conceptualization; formal analysis; funding acquisition; investigation; project administration; writing—original draft. **Chia‐Yang Lin**: Data curation; formal analysis. **Hung‐Pei Tsai**: Investigation; methodology. **Chih‐Lung Lin**: Formal analysis. **Chieh‐Hsin Wu**: Conceptualization; funding acquisition; project administration; supervision; validation; writing—review and editing.

## CONFLICT OF INTEREST STATEMENT

The authors declared no conflicts of interest.

## CONSENT TO PARTICIPATE

All authors approved to participate.

## CONSENT FOR PUBLICATION

All authors approved publication.

### PEER REVIEW

The peer review history for this article is available at https://publons.com/publon/10.1002/brb3.3398.

## Data Availability

All data generated or analyzed during this study are included in this published article and its supplementary information files.

## References

[brb33398-bib-0001] Basu, A. , & Pal, D. (2010). Two faces of protein kinase Cdelta: The contrasting roles of PKCdelta in cell survival and cell death. The Scientific World Journal [Electronic Resource], 10, 2272–2284. 10.1100/tsw.2010.214 21103796 PMC5763792

[brb33398-bib-0002] Borella, R. , Forti, L. , Gibellini, L. , De Gaetano, A. , De Biasi, S. , Nasi, M. , & Pinti, M. (2019). Synthesis and anticancer activity of CDDO and CDDO‐Me, two derivatives of natural triterpenoids. Molecules (Basel, Switzerland), 24(22), 4097. 10.3390/molecules24224097 31766211 PMC6891335

[brb33398-bib-0003] Cao, B. , Scherrer, G. , & Chen, L. (2022). Spinal cord retinoic acid receptor signaling gates mechanical hypersensitivity in neuropathic pain. Neuron, 110(24), 4108–4124. e6. 10.1016/j.neuron.2022.09.027 36223767 PMC9789181

[brb33398-bib-0004] Cesare, P. , Dekker, L. V. , Sardini, A. , Parker, P. J. , & McNaughton, P. A. (1999). Specific involvement of PKC‐epsilon in sensitization of the neuronal response to painful heat. Neuron, 23(3), 617–624. 10.1016/s0896-6273(00)80813-2 10433272

[brb33398-bib-0005] Chen, F. , Chen, F. , Shang, Z. , Shui, Y. , Wu, G. , Liu, C. , & Li, Y. (2017). White matter microstructure degenerates in patients with postherpetic neuralgia. Neuroscience Letters, 656, 152–157. 10.1016/j.neulet.2017.07.023 28729077

[brb33398-bib-0006] Chen, P. , Wang, C. , Ren, Y. N. , Ye, Z. J. , Jiang, C. , & Wu, Z. B. (2021). Alterations in the gut microbiota and metabolite profiles in the context of neuropathic pain. Molecular Brain, 14(1), 50. 10.1186/s13041-021-00765-y 33750430 PMC7941960

[brb33398-bib-0007] Chen, S. P. , Zhou, Y. Q. , Liu, D. Q. , Zhang, W. , Manyande, A. , Guan, X. H. , & Omar, D. M. (2017). PI3K/Akt pathway: A potential therapeutic target for chronic pain. Current Pharmaceutical Design, 23(12), 1860–1868. 10.2174/1381612823666170210150147 28190392

[brb33398-bib-0008] Delaney, A. , Colvin, L. A. , Fallon, M. T. , Dalziel, R. G. , Mitchell, R. , & Fleetwood‐Walker, S. M. (2009). Postherpetic neuralgia: From preclinical models to the clinic. Neurotherapeutics, 6(4), 630–637. 10.1016/j.nurt.2009.07.005 19789068 PMC5084285

[brb33398-bib-0009] Graber, D. J. , Park, P. J. , Hickey, W. F. , & Harris, B. T. (2011). Synthetic triterpenoid CDDO derivatives modulate cytoprotective or immunological properties in astrocytes, neurons, and microglia. Journal of Neuroimmune Pharmacology, 6(1), 107–120. 10.1007/s11481-010-9240-9 20809391

[brb33398-bib-0010] Grossoni, V. C. , Falbo, K. B. , Kazanietz, M. G. , de Kier Joffe, E. D. , & Urtreger, A. J. (2007). Protein kinase C delta enhances proliferation and survival of murine mammary cells. Molecular Carcinogenesis, 46(5), 381–390. 10.1002/mc.20287 17219421

[brb33398-bib-0011] Gruver, C. , & Guthmiller, K. B. (2023). Postherpetic neuralgia. StatPearls Publishing. 29630250

[brb33398-bib-0012] Hains, B. C. , & Waxman, S. G. (2006). Activated microglia contribute to the maintenance of chronic pain after spinal cord injury. Journal of Neuroscience, 26(16), 4308–4317. 10.1523/JNEUROSCI.0003-06.2006 16624951 PMC6674010

[brb33398-bib-0013] Hatch, M. N. , Cushing, T. R. , Carlson, G. D. , & Chang, E. Y. (2018). Neuropathic pain and SCI: Identification and treatment strategies in the 21st century. Journal of the Neurological Sciences, 384, 75–83. 10.1016/j.jns.2017.11.018 29249383

[brb33398-bib-0014] Haydon, P. G. (2001). GLIA: Listening and talking to the synapse. Nature Reviews Neuroscience, 2(3), 185–193. 10.1038/35058528 11256079

[brb33398-bib-0015] He, Y. , & Wang, Z. J. (2015). Nociceptor beta II, delta, and epsilon isoforms of PKC differentially mediate paclitaxel‐induced spontaneous and evoked pain. Journal of Neuroscience, 35(11), 4614–4625. 10.1523/JNEUROSCI.1580-14.2015 25788678 PMC4363388

[brb33398-bib-0016] Hong, B. , Sun, J. , Zheng, H. , Le, Q. , Wang, C. , Bai, K. , He, J. , He, H. , & Dong, Y. (2018). Effect of tetrodotoxin pellets in a rat model of postherpetic neuralgia. Marine Drugs, 16(6), 195. 10.3390/md16060195 29874779 PMC6025269

[brb33398-bib-0017] Hsieh, Y. L. , Chiang, H. , Lue, J. H. , & Hsieh, S. T. (2012). P2×3‐mediated peripheral sensitization of neuropathic pain in resiniferatoxin‐induced neuropathy. Experimental Neurology, 235(1), 316–325. 10.1016/j.expneurol.2012.02.013 22391132

[brb33398-bib-0018] Hsieh, Y. L. , Chiang, H. , Tseng, T. J. , & Hsieh, S. T. (2008). Enhancement of cutaneous nerve regeneration by 4‐methylcatechol in resiniferatoxin‐induced neuropathy. Journal of Neuropathology and Experimental Neurology, 67(2), 93–104. 10.1097/nen.0b013e3181630bb8 18219259

[brb33398-bib-0019] Ji, R. R. , Donnelly, C. R. , & Nedergaard, M. (2019). Astrocytes in chronic pain and itch. Nature Reviews Neuroscience, 20(11), 667–685. 10.1038/s41583-019-0218-1 31537912 PMC6874831

[brb33398-bib-0020] Jin, H. , Kanthasamy, A. , Harischandra, D. S. , Kondru, N. , Ghosh, A. , Panicker, N. , Anantharam, V. , Rana, A. , & Kanthasamy, A. G. (2014). Histone hyperacetylation up‐regulates protein kinase Cdelta in dopaminergic neurons to induce cell death: Relevance to epigenetic mechanisms of neurodegeneration in Parkinson disease. Journal of Biological Chemistry, 289(50), 34743–34767. 10.1074/jbc.M114.576702 25342743 PMC4263877

[brb33398-bib-0021] Kierdorf, K. , & Prinz, M. (2017). Microglia in steady state. Journal of Clinical Investigation, 127(9), 3201–3209. 10.1172/JCI90602 28714861 PMC5669563

[brb33398-bib-0022] Kim, Y. H. , Kim, Y. S. , Park, C. H. , Chung, I. Y. , Yoo, J. M. , Kim, J. G. , & Choi, W. S. (2008). Protein kinase C‐delta mediates neuronal apoptosis in the retinas of diabetic rats via the Akt signaling pathway. Diabetes, 57(8), 2181–2190. 10.2337/db07-1431 18443201 PMC2494683

[brb33398-bib-0023] Kohno, K. , Kitano, J. , Kohro, Y. , Tozaki‐Saitoh, H. , Inoue, K. , & Tsuda, M. (2018). Temporal kinetics of microgliosis in the spinal dorsal horn after peripheral nerve injury in rodents. Biological & Pharmaceutical Bulletin, 41(7), 1096–1102. 10.1248/bpb.b18-00278 29962405

[brb33398-bib-0024] Lei, Y. , Sun, Y. , Lu, C. , Ma, Z. , & Gu, X. (2016). Activated glia increased the level of proinflammatory cytokines in a resiniferatoxin‐induced neuropathic pain rat model. Regional Anesthesia and Pain Medicine, 41(6), 744–749. 10.1097/AAP.0000000000000441 27429048

[brb33398-bib-0025] Leo, M. , Schulte, M. , Schmitt, L. I. , Schafers, M. , Kleinschnitz, C. , & Hagenacker, T. (2017). Intrathecal resiniferatoxin modulates TRPV1 in DRG neurons and reduces TNF‐induced pain‐related behavior. Mediators of Inflammation, 2017, 2786427. 10.1155/2017/2786427 28831207 PMC5558708

[brb33398-bib-0026] Li, N. Q. , Peng, Z. , Xu, W. W. , An, K. , & Wan, L. (2021). Bone mesenchymal stem cells attenuate resiniferatoxin‐induced neuralgia via inhibiting TRPA1‐PKCdelta‐P38/MAPK‐p‐P65 pathway in mice. Brain Research Bulletin, 174, 92–102. 10.1016/j.brainresbull.2021.06.004 34098041

[brb33398-bib-0027] Lin, C. L. , Fu, Y. S. , Hsiao, T. H. , & Hsieh, Y. L. (2013). Enhancement of purinergic signalling by excessive endogenous ATP in resiniferatoxin (RTX) neuropathy. Purinergic Signalling, 9(2), 249–257. 10.1007/s11302-012-9347-y 23264185 PMC3646125

[brb33398-bib-0028] Liu, Q. , Qiu, J. , Liang, M. , Golinski, J. , van Leyen, K. , Jung, J. E. , & Whalen, M. J. (2014). Akt and mTOR mediate programmed necrosis in neurons. Cell Death & Disease, 5, e1084. 10.1038/cddis.2014.69 24577082 PMC3944276

[brb33398-bib-0029] Milligan, E. D. , & Watkins, L. R. (2009). Pathological and protective roles of glia in chronic pain. Nature Reviews Neuroscience, 10(1), 23–36. 10.1038/nrn2533 19096368 PMC2752436

[brb33398-bib-0030] Oaklander, A. L. (2008). Mechanisms of pain and itch caused by herpes zoster (shingles). The Journal of Pain, 9(1), S10–S18. 10.1016/j.jpain.2007.10.003 18166461

[brb33398-bib-0031] Ohtori, S. , Takahashi, K. , Moriya, H. , & Myers, R. R. (2004). TNF‐alpha and TNF‐alpha receptor type 1 upregulation in glia and neurons after peripheral nerve injury: Studies in murine DRG and spinal cord. Spine (Phila Pa 1976), 29(10), 1082–1088. 10.1097/00007632-200405150-00006 15131433

[brb33398-bib-0032] Pan, H. L. , Khan, G. M. , Alloway, K. D. , & Chen, S. R. (2003). Resiniferatoxin induces paradoxical changes in thermal and mechanical sensitivities in rats: Mechanism of action. Journal of Neuroscience, 23(7), 2911–2919. 12684478 10.1523/JNEUROSCI.23-07-02911.2003 PMC6742104

[brb33398-bib-0033] Phillips, D. R. , Rasbery, J. M. , Bartel, B. , & Matsuda, S. P. (2006). Biosynthetic diversity in plant triterpene cyclization. Current Opinion in Plant Biology, 9(3), 305–314. 10.1016/j.pbi.2006.03.004 16581287

[brb33398-bib-0034] Robles, L. , Vaziri, N. D. , Li, S. , Masuda, Y. , Takasu, C. , Takasu, M. , & Ichii, H. (2016). Synthetic triterpenoid RTA dh404 (CDDO‐dhTFEA) ameliorates acute pancreatitis. Pancreas, 45(5), 720–729. 10.1097/MPA.0000000000000518 26495793 PMC5847282

[brb33398-bib-0035] Schmader, K. (2018). Herpes zoster. Annals of Internal Medicine, 169(3), ITC19–ITC31. 10.7326/AITC201808070 30083718

[brb33398-bib-0036] Schutzer‐Weissmann, J. , & Farquhar‐Smith, P. (2017). Post‐herpetic neuralgia—A review of current management and future directions. Expert Opinion on Pharmacotherapy, 18(16), 1739–1750. 10.1080/14656566.2017.1392508 29025327

[brb33398-bib-0037] Suh, N. , Wang, Y. , Honda, T. , Gribble, G. W. , Dmitrovsky, E. , Hickey, W. F. , & Sporn, M. B. (1999). A novel synthetic oleanane triterpenoid, 2‐cyano‐3,12‐dioxoolean‐1,9‐dien‐28‐oic acid, with potent differentiating, antiproliferative, and anti‐inflammatory activity. Cancer Research, 59(2), 336–341. 9927043

[brb33398-bib-0038] Sun, R. Q. , Tu, Y. J. , Yan, J. Y. , & Willis, W. D. (2006). Activation of protein kinase B/Akt signaling pathway contributes to mechanical hypersensitivity induced by capsaicin. Pain, 120(1–2), 86–96. 10.1016/j.pain.2005.10.017 16360265

[brb33398-bib-0039] Tran, T. A. , McCoy, M. K. , Sporn, M. B. , & Tansey, M. G. (2008). The synthetic triterpenoid CDDO‐methyl ester modulates microglial activities, inhibits TNF production, and provides dopaminergic neuroprotection. Journal of Neuroinflammation, 5, 14. 10.1186/1742-2094-5-14 18474101 PMC2396606

[brb33398-bib-0040] Velazquez, K. T. , Mohammad, H. , & Sweitzer, S. M. (2007). Protein kinase C in pain: Involvement of multiple isoforms. Pharmacological Research, 55(6), 578–589. 10.1016/j.phrs.2007.04.006 17548207 PMC2140050

[brb33398-bib-0041] Watson, C. P. N. , Deck, J. H. , Morshead, C. , Van der Kooy, D. , & Evans, R. J. (1991). Post‐herpetic neuralgia: Further post‐mortem studies of cases with and without pain. Pain, 44(2), 105–117. 10.1016/0304-3959(91)90124-G 1711192

[brb33398-bib-0042] Werner, R. N. , Nikkels, A. F. , Marinovic, B. , Schafer, M. , Czarnecka‐Operacz, M. , Agius, A. M. , & Nast, A. (2017). European consensus‐based (S2k) Guideline on the Management of Herpes Zoster—Guided by the European Dermatology Forum (EDF) in cooperation with the European Academy of Dermatology and Venereology (EADV), Part 1: Diagnosis. Journal of the European Academy of Dermatology and Venereology, 31(1), 9–19. 10.1111/jdv.13995 27804172

[brb33398-bib-0043] Wright, D. M. , Small, K. M. , Nag, S. , & Mokha, S. S. (2019). Activation of membrane estrogen receptors attenuates NOP‐mediated tactile antihypersensitivity in a rodent model of neuropathic pain. Brain Sciences, 9(6), 147. 10.3390/brainsci9060147 31234278 PMC6628583

[brb33398-bib-0044] Wu, C. H. , Lv, Z. T. , Zhao, Y. , Gao, Y. , Li, J. Q. , Gao, F. , & Li, M. (2013). Electroacupuncture improves thermal and mechanical sensitivities in a rat model of postherpetic neuralgia. Molecular Pain, 9, 18. 10.1186/1744-8069-9-18 23551937 PMC3626545

[brb33398-bib-0045] Wu, C. H. , Wu, M. K. , Lu, C. C. , Tsai, H. P. , Lu, Y. Y. , & Lin, C. L. (2021). Impact of hepatoma‐derived growth factor blockade on resiniferatoxin‐induced neuropathy. Neural Plasticity, 2021, 8854461. 10.1155/2021/8854461 33727914 PMC7937473

[brb33398-bib-0046] Xu, B. , Mo, C. , Lv, C. , Liu, S. , Li, J. , Chen, J. , & Guan, X. (2019). Post‐surgical inhibition of phosphatidylinositol 3‐kinase attenuates the plantar incision‐induced postoperative pain behavior via spinal Akt activation in male mice. BMC Neuroscience [Electronic Resource], 20(1), 36. 10.1186/s12868-019-0521-9 31366324 PMC6668088

[brb33398-bib-0047] Yuan, X. C. , Wu, C. H. , Gao, F. , Li, H. P. , Xiang, H. C. , Zhu, H. , & Li, M. (2017). Activation and expression of mu‐calpain in dorsal root contributes to RTX‐induced mechanical allodynia. Molecular Pain, 13, 1744806917719169. 10.1177/1744806917719169 28714350 PMC5548329

[brb33398-bib-0048] Zhao, T. , Ding, Y. , Li, M. , Zhou, C. , & Lin, W. (2019). Silencing lncRNA PVT1 inhibits activation of astrocytes and increases BDNF expression in hippocampus tissues of rats with epilepsy by downregulating the Wnt signaling pathway. Journal of Cellular Physiology, 234(9), 16054–16067. 10.1002/jcp.28264 30805931

[brb33398-bib-0049] Zorzoli, E. , Pica, F. , Masetti, G. , Franco, E. , Volpi, A. , & Gabutti, G. (2018). Herpes zoster in frail elderly patients: Prevalence, impact, management, and preventive strategies. Aging Clinical and Experimental Research, 30(7), 693–702. 10.1007/s40520-018-0956-3 29721782

